# Consuming Parasitized Aphids Alters the Life History and Decreases Predation Rate of Aphid Predator

**DOI:** 10.3390/insects11120889

**Published:** 2020-12-17

**Authors:** Jian-Feng Liu, Xiu-Qin Wang, Jacqueline R. Beggs, Hou-Ding Ou, Xiao-Fei Yu, Xiu-Xian Shen, Mao-Fa Yang

**Affiliations:** 1 State Key Laboratory Breeding Base of Green Pesticide and Agricultural Bioengineering, Key Laboratory of Green Pesticide and Agricultural Bioengineering, Ministry of Education, Guizhou University, Guiyang 550025, China; jfliu3@gzu.edu.cn; 2 Institute of Entomology, Guizhou University, Guizhou Provincial Key Laboratory for Agricultural Pest Management of the Mountainous Region, Scientific Observing and Experimental Station of Crop Pest in Guiyang, Ministry of Agriculture, Guiyang 550025, China; xiuqinw@yeah.net (X.-Q.W.); ouhouding@126.com (H.-D.O.); xiuxianshen_2017@126.com (X.-X.S.); 3 Centre for Biodiversity and Biosecurity, School of Biological Sciences, The University of Auckland, Auckland 1072, New Zealand; j.beggs@auckland.ac.nz; 4 College of Tobacco Sciences, Guizhou University, Guiyang 550025, China; xfyu1@gzu.edu.cn

**Keywords:** intraguild predation, *Aphidoletes aphidimyza* (Rondani), *Aphidius gifuensis* (Ashmead), *Myzus persicae* (Sulzer), age-stage two-sex life table

## Abstract

**Simple Summary:**

Intraguild predation is a common phenomenon between predators and parasitoids. Despite numerous studies on the performance of intraguild predators by consuming on intraguild prey, the entire two-sex life table and predation rates of intraguild predators fed on intraguild prey remain poorly known. In this study, we investigated the effect of parasitized *Myzus persicae* aphids by *Aphidius gifuensis* (Ashmead) on the entire two-sex life table and predation rates of *Aphidoletes aphidimyza* (Rondani). Our results showed that feeding on parasitized aphids did not influence the survival rates of immature *A. aphidimyza* individuals but significantly increased the development time of *A. aphidimyza* individuals and markedly reduced their longevity. The predation rate of immature *A. aphidimyza* individuals was also adversely affected by feeding on parasitized aphids. These results provide basic data for the potential use of *A. aphidimyza* in combination with *A. gifuensis* in *M. persicae* control programs.

**Abstract:**

Intraguild predation interactions have substantial theoretical and practical implications for the dynamics of natural competitor populations used for biological control. Intraguild predation on parasitized aphids not only has a direct, negative effect on the parasitoid species, but it may indirectly influence the predator’s development, survival, reproduction and predation rates. In this study, we used two-sex life table theory, life table parameters and predation rates of *Aphidoletes aphidimyza* (Rondani) to compare when its populations fed on aphids (*Myzus persicae* Sulzer) (Hemiptera: Aphididae) that were either unparasitized or parasitized by *Aphidius gifuensis* (Ashmead) (Hymenoptera: Braconidae). Our results showed that individuals of *A. aphidimyza* were capable of completing their development and attaining maturity when they fed on parasitized aphids. Although feeding on parasitized aphids did not influence the survival rates of immature *A. aphidimyza*, it did significantly slow their development and extended their longevity, thereby reducing the fecundity and predation rates of *A. aphidimyza*. These findings may be pivotal for better understanding the sustained coexistence of predators with parasitoids in the biological control of aphids.

## 1. Introduction

Intraguild predation (IGP) arises when natural competitors of a shared resource also engage in predation or parasitism in food webs of prey and their natural enemies at their same trophic level [1,2]. A common case of IGP interaction within aphidophagous guild involves three species: A shared resource (aphids), a predator of the aphid such as a predatory midge or coccinellid beetle [3], and a parasitoid of the aphid. IGP also occurs when the predator consumes the parasitoid offspring developing in the aphid. In this respect, IGP may have the potential to greatly disrupt the distribution, abundance, evaluation, and control efficiency of predators [1]. Predatory brown lacewing *Micromus variegatus* (Fabricius) and lady beetle *Coccinella septempunctata* L. prefers to consume parasitized aphids over unparasitized aphids [4,5]. However, the consumption of intraguild prey by the intraguild predator can also negatively affect the development, survival, and oviposition of such predators [6,7,8,9,10]. Conversely, under greenhouse conditions, the combination of *M. variegatus* and *Aphidius ervi* Haliday did not result in intraguild predator densities and shared prey compared with intraguild predator alone [4]. The simultaneous application of predators and parasitoids could improve the efficacy of aphid control under greenhouse operations [11,12]. Yet, there is little information about how parasitism of aphids may affect the predator’s life history or its predation rates.

The green peach aphid, *Myzus persicae* (Sulzer) (Hemiptera: Aphididae), is the most devastating pest aphid worldwide, capable of attacking more than 400 plant species, including key Solanaceae, Cruciferae, and Leguminosae crops [13,14]. This aphid species have a short lifespan, rapid reproduction, and causes significant damage to plants by injecting viruses into them, which causes severe losses in crop production yields [15,16]. *Myzus persicae* can directly extract sap from young leaves and excretes honeydew, which contaminates foliage and fruit and induces sooty mold [17]. *Aphidoletes aphidimyza* (Rondani), aphidophagous gall midge that can attack more than 80 aphid species [18]. Third instar larvae of *A. aphidimyza* can kill 4–50 *M. persicae* nymphs per day [19]. Releasing *A. aphidimyza* pupae in a predator:aphid ratio (1:10 and 1:15) causes exceeded 95% loss rates of *M. persicae* nymphs on tobacco in the field [20]. The control efficacy of *A. aphidimyza* on aphids appears high when implemented with parasitoids [4]. Another biocontrol agent of aphids, the solitary endoparasitoid *Aphidius gifuensis* (Ashmead) (Hymenoptera: Braconidae), has been extensively used as a biocontrol agent against *M. persicae* in China [21,22,23]. Notably, *A. gifuensis* has been successfully mass-reared and incrementally released to control *M. persicae* populations in fields of tobacco and vegetables [16]. Both *A. gifuensis* and *A. aphidimyza* often co-occur in host-crop fields, and the latter can easily replace colonies of *A. gifuensis* on tobacco in greenhouses in Leshan Town of Zunyi City in Guizhou Province, China (personal observations). The IGP interaction between this predatory midge and parasitoid is unidirectional, with *A. aphidimyza* killing parasitoids [24]. *Aphidoletes aphidimyza* ate aphids parasitized by *Aphidius colemani* Viereck and thereby reduced the production of parasitoids [25]. Intraguild interactions are mostly detrimental to the aphid parasitoid population [24]. However, it remains unclear whether these ecological interactions could adversely influence the life history and consumption of *A. aphidimyza* populations.

Life table analysis can provide comprehensive information on population growth rates, and life table parameters are important tools for comparing the fitness of populations and understanding the effects of external factors on fitness (e.g., temperature or diet) [26,27]. Chi and Liu (1985) replaced the traditional female life table with the age-staged two-sex life table, which allows for the projection of populations and provides a clearer division of life history phases than classical life tables [28]. To date, however, no previous studies have investigated the effect of parasitized aphids on two-sex life tables and predation rates to assess the net effect on the predator population.

Previous studies showed that *A. aphidimyza* has a negative preference for parasitoid mummies [11]. We hypothesized that feeding on young parasitized aphids also would adversely affect *A. aphidimyza*’s survival, development, reproduction, and predation rate. Therefore, in this study, we chose young parasitized aphids to understand their effects on life history and rate of predation by *A. aphidimyza* to provide information for the simultaneous application of two biocontrol agents in the greenhouse.

## 2. Materials and Methods

### 2.1. Insect Colonies

The *M. persicae* aphids were collected from tobacco (*Nicotiana tabacum*) leaves at the Natural Enemy Breeding Center, in Jinhua Town of Fenggang County (Guizhou Province, China), on 1 May 2017. The reared colonies were kept on tobacco (“K326”) plants in mesh cages (45 cm × 45 cm × 30 cm) in the greenhouse belonging to the Institute of Entomology, Guizhou University.

The *A. gifuensis* parasitoids were originally obtained from *M. persicae* in a tobacco farm located in Leshan Town (Zunyi City, Guizhou Province), and then, in a greenhouse, reared on *M. persicae* as a host for more than five generations. We transferred more than 400 *M. persicae* onto tobacco plants enclosed by synthetic fine-nylon mesh cages, after which we introduced 30–40 pairs of *A. gifuensis* adults into each cage at 25 °C ± 1 °C; 24 h later, we removed these adults from the mesh cages, and young parasitized fourth instar *M. persicae* nymphs were prepared for experiments 2 days later. The young parasitized aphids moved very slowly, and their abdomen backs were lightly colored. The percentage of parasitized aphids exceeded over 98% in the experiment.

We obtained the *A. aphidimyza* larval predators from the same location as *A. gifuensis*. *Aphidoletes aphidimyza* was reared with *M. persicae* aphids on tobacco for three generations in an artificial climate chamber. Thirty pairs of *A. aphidimyza* adults were allowed to oviposit on fresh and healthy tobacco plants with enough *M. persicae* aphids for one day in a mesh cage (45 cm × 45 cm × 30 cm). After all *A. aphidimyza* adults were removed from the plant, *A. aphidimyza* eggs were prepared for the experiment. All experiments were conducted in an artificial climate chamber at 25 °C ± 1 °C, 65% ± 5% relative humidity (RH), with a 14-L:10-D photoperiod.

### 2.2. Life Table Analyses

To evaluate the effect of parasitized *M. persicae* on life-history parameters and predation rates of *A. aphidimyza*, we maintained a total of 52 freshly laid eggs (<12 h) by *A. aphidimyza* females in modified Petri dishes (35 mm-diameter) as described above, for each treatment (parasitized vs. unparasitized aphids as food). We recorded the incubation rate of eggs daily until the larvae hatched.

Based on a previous study of the predation ability of *A. aphidimyza* on *M. persicae* [19], each hatched larva was provided daily with 30 unparasitized or parasitized fourth instar *M. persicae*. We observed the larvae every day and determined developmental time and survival rate. We also counted the number of unparasitized and parasitized *M. persicae* consumed by *A. aphidimyza* daily before pupation. When *A. aphidimyza* reached the third instar stage of development, we placed balls of moist cotton on each Petri dish; mature larvae were able to access this moist absorbent cotton for their pupation. We sprayed distilled water daily onto cotton balls where the pupae were developing to keep the cotton moist until the emergence of adults. Newly emerged male and female *A. aphidimyza* were paired and moved into an artificial fine-nylon mesh cage (45 cm × 45 cm× 30 cm), which contained a tobacco seedling with 120 unparasitized or parasitized fourth instar *M. persicae* nymphs. New tobacco seedlings with infected aphids (as described above) and absorbent cotton soaked in a 10% honey solution were provided daily for these *A. aphidimyza* adults. We recorded the preoviposition period, oviposition period, fecundity, and longevity of *A. aphidimyza* individuals until all of them had died.

### 2.3. Life Table Data Analysis

For the age-staged life table, age-stage specific survival rate (*S_xj_*), female age-specific fecundity (*f_x6_*), age-specific maternity (*l_x_m_x_*), age-specific survival rate (*l_x_*) and age-specific fecundity of the total population (*m_x_*) of *A. aphidimyza* were calculated using the program TWOSEX-MSChart [29,30]. Mean values and standard errors of developmental time, survival rate, adult preoviposition period, total preoviposition period (calculated from birth to oviposition), oviposition period, and fecundity of *A. aphidimyza*, as well its predation rates, were estimated with 10,000 bootstrap replicates. A paired bootstrap test was used to calculate life table parameters of *A. aphidimyza* when it fed on unparasitized or parasitized *M. persicae* [31]. Age-stage-specific consumption (*c_xj_*), age-specific predation rate (*k_x_*), age-specific net predation rate (*q_x_*), net predation rate (*C*_0_), stable predation rate (*ψ*), finite predation rate (*λ*) and transformation rate (*Q_p_*) of *A. aphidimyza* were analyzed according to the CONSUME-MSChart program [29,32]. We used the TIMING-MSChart program [29,33] to project the population growth, stage growth, total population and total consumption of *A. aphidimyza* reared on parasitized or unparasitized *M. persicae*. We used Sigma Plot v12.5 to construct the curves for *A. aphidimyza*’s survival rates, reproductive value, and predation rates.

## 3. Results

### 3.1. Effect of Parasitized Aphids on the Survival of A. aphidimyza

**Figure 1:**
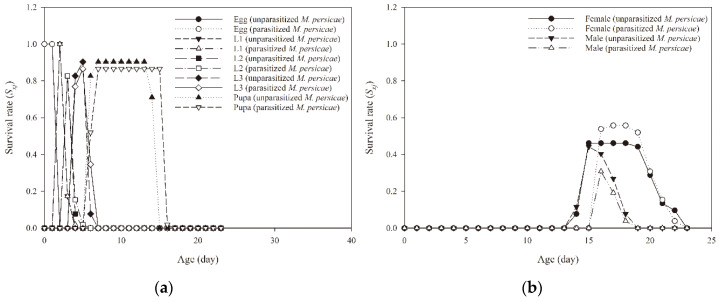
Age-stage specific survival rate (*S_xj_*) of immature (**a**) and adult (**b**) *Aphidoletes aphidimyza* that fed on unparasitized and parasitized *Myzus persicae* by *Aphidius gifuensis.*

**Figure 2:**
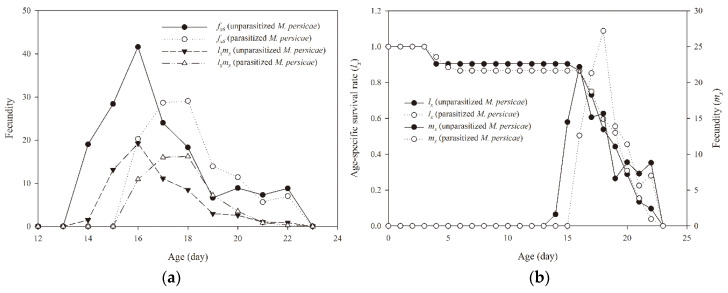
Female age-specific fecundity (*f_x6_*, (**a**)), age-specific maternity (*l_x_m_x_*, (**a**)), age-specific survival rate (*l_x_*, (**b**)) and age-specific fecundity of the total population (*m_x_*, (**b**)) of *Aphidoletes aphidimyza* that fed on unparasitized and parasitized *Myzus persicae* by *Aphidius gifuensis.*

Feeding on parasitized aphids did not influence the survival rates of immature *A. aphidimyza* individuals (Table 1). Of the 52 *A. aphidimyza* eggs initially collected from the prepared colonies, 47 and 45 individuals developed into adults when feeding on unparasitized and parasitized *M. persicae*, respectively. The age-stage specific survival rate (*S*_xj_) of individual immature stages of *A. aphidimyza* overlapped between unparasitized and parasitized *M. persicae* (Figure 1a). Age-specific survival rate (*l_x_*) of *A. aphidimyza* showed a similar trend when it fed on unparasitized and parasitized *M. persicae* (Figure 2b).

### 3.2. Effect of Parasitized Aphids on Developmental Time, Longevity, and Fecundity of A. aphidimyza

Feeding on *M. persicae* parasitized by *A. gifuensis* significantly increased the development time of *A. aphidimyza* individuals and markedly reduced their longevity (Table 1). Third instar larvae, as well as pupae and the total duration of the immature stage *A. aphidimyza* that fed on unparasitized *M. persicae,* developed markedly faster than those arising when the predator fed on aphids parasitized by *A. gifuensis.* Female and male *A. aphidimyza* had significantly shorter lifespans when they fed on parasitized than unparasitized *M. persicae.* However, there was no significant difference in the adult preoviposition period (APOP) of *A. aphidimyza* feeding on parasitized and unparasitized *M. persicae*. The total preoviposition period (TPOP) of *A. aphidimyza* was significantly longer for those that fed on parasitized than unparasitized *M. persicae.* Recorded oviposition periods were longer, and fecundity was markedly higher of *A. aphidimyza* that fed on unparasitized *M. persicae* than the *A. aphidimyza* that consumed parasitized *M. persicae* (Table 1). The maximum peak of female age-specific fecundity (*f_x6_ =* 41.58) and maximal age-specific maternity (*l_x_m_x_ =* 19.19) of *A. aphidimyza* occurred at 16 days of age when feeding on unparasitized *M. persicae*; however, age-specific fecundity showed a later maximal peak (*m_x_*), at 18 days, when parasitized *M. persicae* were the prey (Figure 2).

### 3.3. Population Parameters

The net reproductive rate (*R*_0_), intrinsic rate of natural increase (*r_m_*), and finite rate of increase (*λ*) of *A. aphidimyza* was similar between predator populations feeding on unparasitized and parasitized *M. persicae*. Nevertheless, the mean generation time (*T*) of *A. aphidimyza* was significantly higher when it fed on parasitized than unparasitized *M. persicae* aphids (Table 2).

### 3.4. Predation Rate

**Figure 3:**
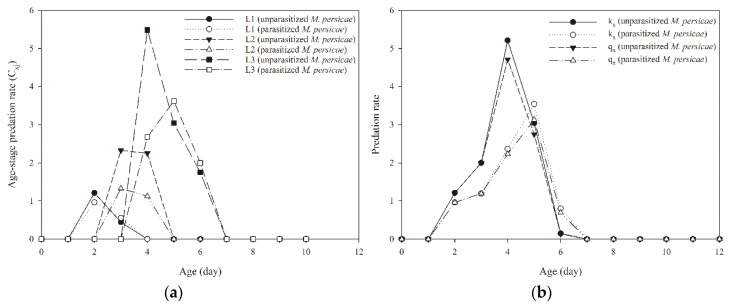
Age-stage, two-sex predation rate (*c_xj_*) (**a**) and age-specific predation rate (*k_x_*) (**b**), age-specific net predation rate (*q_x_*) (**b**) of immature *Aphidoletes aphidimyza* that fed on unparasitized and parasitized *Myzus persicae* by *Aphidius gifuensis.*

Parasitized *M. persicae* significantly affected the rates of predation by immature *A. aphidimyza* individuals (Table 3). Both immature female and male *A. aphidimyza* consumed more unparasitized *M. persicae* than those that fed on parasitized *M. persicae.* The age-stage-specific predation rate (*C_xj_*) of *A. aphidimyza* was strongly affected by parasitized *M. persicae* as prey, especially in the second and third larval stages (Figure 3a). The maximum peak of the age-stage-specific predation rate (*C_xj_*) of *A. aphidimyza* occurred at the age of 4 d when the predator fed on unparasitized *M. persicae.* Age-specific predation rate (*k_x_*) and age-specific net predation rate (*q*_x_) curves of *A. aphidimyza* were both higher when unparasitized aphids were eaten instead of parasitized ones (Figure 3b). Taking survival rate and longevity of *A. aphidimyza* into account, feeding on parasitized *M. persicae* significantly reduced *A. aphidimyza*’s net predation rate (*C*_0_), stable predation rate (*ψ*), and finite predation rate (*λ*) when compared with its feeding on unparasitized *M. persicae.* However, eating parasitized *M. persicae* did not affect the transformation rate (*Q_p_*) of *A. aphidimyza* (Table 3).

### 3.5. Population Projection

**Figure 4:**
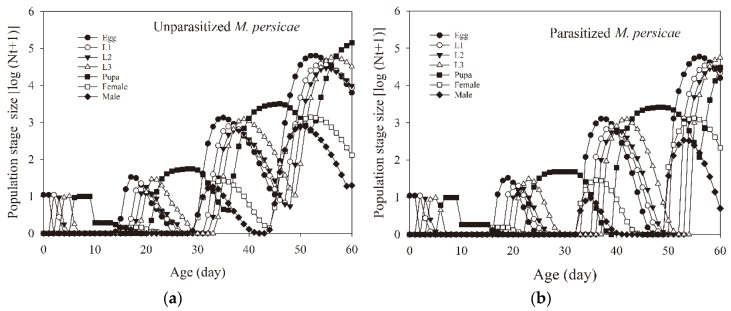
Population growth of *Aphidoletes aphidimyza* fed on unparasitized *Myzus persicae* (**a**) and parasitized *Myzus persicae* (**b**) by computer simulation.

**Figure 5:**
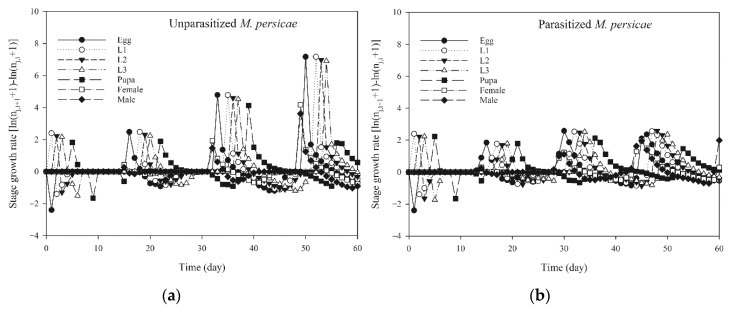
Stage population growth rate of *Aphidoletes aphidimyza* fed on unparasitized *Myzus persicae* (**a**) and parasitized *Myzus persicae* (**b**) by computer simulation.

**Figure 6:**
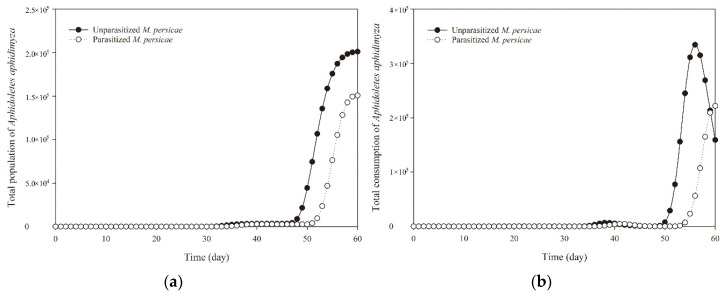
Total population (**a**) and total consumption (**b**) of *Aphidoletes aphidimyza* that fed on unparasitized and parasitized *Myzus persicae* by *Aphidius gifuensis.*

To evaluate the effect of parasitized *M. persicae* prey on the population stages’ size and growth rate of *A. aphidimyza* as well as its predation rates, the data from a two-sex life table and for predation rates were used together to project the growth of *A. aphidimyza* population and its consumption of aphids (Figure 4, Figure 5 and Figure 6). The total population of *A. aphidimyza* feeding on unparasitized aphids at 60 days reached 201,113 individuals, which was higher than those predators the prediction obtained at 60 days (150,816 individuals) for *A. aphidimyza* that fed on parasitized aphids (Figure 6a). Moreover, those *A. aphidimyza* individuals feeding on unparasitized aphids also featured higher predation ability with those feeding on parasitized aphids at 56 days (Figure 6b). Since predators only have predatory capacity during the larval stage, these consumption values omitted pupal and adult periods.

## 4. Discussion

Consumption of parasitized aphids and mummies could adversely affect the development, body size, survival, and behavior of aphid predators [6,7,10,34,35]. Our results showed that consumption of parasitized aphids did not influence the survival rates of immature *A. aphidimyza* individuals, but it did significantly delay their overall developmental time and also reduced the predator’s longevity, fecundity and predation rates. To our best knowledge, this study is the first to have comprehensively evaluated the effect of parasitized aphids on the entire life history and predation rates of aphid predators.

The survival rates of immature *A. aphidimyza* were not significantly influenced by feeding on parasitized *M. persicae*, and over 90% of the predator egg survived to adult emergence. Similar results were reported for *Harmonia axyridis* and *Propylea japonica* when each fed on *Aphid craccivora* parasitized and mummified by *A. colemani* [34], and also for *Coccinella undecimpunctata* that fed on “injured” *M. persicae* mummies parasitized by *A. colemani* [35]. Compared with parasitized aphids, it seems that mummies do not provide enough nutrition to sustain the development of either *C. septempunctata* or *H. convergens* into adulthood [7,35].

Development times for each stage and total immature stage of *A. aphidimyza* that fed on parasitized *M. persicae* were longer than those of predator larvae feeding on unparasitized *M. persicae.* Our results are consistent with studies of *C. septempunctata*, *H. convergens*, *H. axyridis*, and *P. japonica* that subsisted on mummified aphids, demonstrating that predators could slow their ontogenetic development [7,10,34]. Our study using *A. aphidimyza* revealed that its longevity, oviposition period, and fecundity were all significantly affected by its feeding on parasitized aphids. Little research has been conducted on the effects of parasitized aphid species on the reproduction parameters of their predators. When fed *A. gifuensis* mummies, aphid enemy *H. axyridis* females had a longer pre-oviposition period and did not lay eggs within 30 days of observation [10]. The number of eggs produced by *Episyrphus balteatus* females was affected by mummified aphids and the exuvia of mummies of *Acyrthosiphon pisum*, but the number of eggs laid by *E. balteatus* was nonetheless similar when the female fed with healthy aphids and parasitized aphids [36]. Under a greenhouse choice experiment, *E. balteatus* females laid significantly fewer eggs on mummified *M. persicae* colonies parasitized by *A. colemani* than they do on colonies with unparasitized aphids [37]. Mummies are inferior prey for aphid predators; hence, a qualitative difference in the nutrition between unparasitized and parasitized aphids might influence the development and reproduction of their predators [10,34,35]. Furthermore, the fatty acid content of parasitized cotton aphids could decrease for three days post-parasitization [38]. Compared with the unparasitized aphids, mummies (parasitized ≥9 days) with a dark spot in the abdomen tend to be carbohydrate-poor and richer in proteins and lipids [6,10]. The changed nutritional content of parasitized aphids may be a factor affecting the development and reproduction of predators.

The predation potential of aphid predators is markedly influenced when they feed on parasitized aphids [6,7]. Fourth instar larvae and adults of *H. axyridis* try to consume more unparasitized aphids than mummies parasitized by *A. asychis* in 1 h and 3 h [6]. Compared with unparasitized *A. craccivora*, the larvae of *H. axyridis*, *P. japonica* and *C. septempunctata* consume less parasitized and mummified aphids [34]. However, no previous study has investigated the effects of parasitized aphids upon the daily rate of predation by aphid predators. Our study is the first to demonstrate that the consumption of parasitized aphids can significantly lower the net predation rate, the stable predation rate, and the finite predation rate of *A. aphidimyza*, leading to a weaker predation ability than for those feeding on unparasitized aphids across population projections.

## 5. Conclusions

In conclusion, *A. aphidimyza* could complete its development if feeding solely on parasitized aphids, but it had a longer larval stage development and shorter longevity, longer preoviposition period, and lower reproduction than when feeding on unparasitized aphids. The completed development and reproduction of the predator when feeding on unparasitized and parasitized aphids are both pivotal for maintaining its coexistence with the parasitoid and effective suppression of the aphid in the ecosystem.

## Figures and Tables

**Table 1:** The survival rates, development time (mean ± SE) of various life stages and sexes, reproduction parameters of *Aphidoletes aphidimyza* that fed on unparasitized and parasitized *Myzus persicae* by *Aphidius gifuensis.*

Parameters	Stage	Unparasitized *M. persicae*		Parasitized *M. persicae*		*p*-Value
		N	Mean ± SE	N	Mean ± SE	
	Egg	52	100.0 ± 0	52	100.0 ± 0	-
	1st instar larva	47	92.1 ± 3.7	48	90.4 ± 4.1	0.7276
Survival rates (%)	2nd instar larva	47	100.0 ± 0	45	93.8 ± 3.5	0.0909
	3rd instar larva	47	100.0 ± 0	45	100.0 ± 0	-
	Pupa	47	100.0 ± 0	45	100.0 ± 0	-
	Proportion of female N_f_/N (%)		46.2 ± 6.9		55.8 ± 6.8	0.3178
Development time (d)	Egg	52	2.0 ± 0.0	52	2.0 ± 0.0	-
	1st instar larva	47	1.1 ± 0.0	48	1.1 ± 0.0	0.7775
	2nd instar larva	47	1.0 ± 0.0	45	1.0 ± 0.0	0.5524
	3rd instar larva	47	2.0 ± 0.0	45	2.3 ± 0.1	<0.001
	Pupa	47	8.7 ± 0.1	45	9.6 ± 0.1	<0.001
	Total immature stages	47	14.8 ± 0.1	45	16.0 ± 0.0	<0.001
Reproduction parameters	Female adult longevity (d)	24	6.3 ± 0.3	29	4.8 ± 0.2	<0.001
	Male adult longevity (d)	23	3.0 ± 0.2	16	1.8 ± 0.2	<0.001
	Adult preoviposition period (APOP, d)	24	0.3 ± 0.1	29	0.7 ± 0.5	0.2692
	Total preoviposition period (TPOP, d)	24	15.2 ± 0.1	29	16.6 ± 0.2	<0.001
	Oviposition period (d)	24	5.4 ± 0.3	29	3.8 ± 0.3	<0.001
	Fecundity (eggs per female)	24	131.3 ± 5.9	29	98.5 ± 5.8	<0.001

Significant difference within each row calculated by paired bootstrap test with 10,000 replications.

**Table 2:** Population parameters (means ± SE) of *Aphidoletes aphidimyza* fed on unparasitized and parasitized *Myzus persicae* by *Aphidius gifuensis.*

Parameter	*M. persicae*	Parasitized *M. persicae*	*p*-Value
Net reproduction rate, *R*_0_ (offspring/individual)	60.58 ± 9.46	54.94 ± 7.54	0.6427
Intrinsic rate of increase, *r_m_* (d^−1^)	0.24 ± 0.01	0.22 ± 0.01	0.1202
Finite rate of increase, *λ* (d^−1^)	1.27 ± 0.01	1.24 ± 0.01	0.1197
Mean generation time, *T* (d)	17.40 ± 0.11	18.48 ± 0.12	<0.001

Significant difference within each row calculated by paired bootstrap test (TWOSEX-MSChart; *p* < 0.05).

**Table 3:** Predation rate (aphids/day) by *Aphidoletes aphidimyza* and calculated parameters on unparasitized and parasitized *Myzus persicae* by *Aphidius gifuensis*.

Sex/Stage	Unparasitized *M. persicae*	Parasitized *M. persicae*	*p*-Value
Female			
L1	1.29 ± 0.09	1.00 ± 0.00	0.0023
L2	2.38 ± 0.12	1.41 ± 0.12	<0.001
L3	8.62 ± 0.41	7.10 ± 0.34	0.0036
Male			
L1	1.35 ± 0.12	1.19 ± 0.10	0.2941
L2	2.26 ± 0.21	1.38 ± 0.12	0.0006
L3	7.78 ± 0.33	6.25 ± 0.48	0.0079
Net predation rate, *C*_0_	10.8077 ± 0.5182	8.2115 ± 0.4285	<0.001
Stable predation rate, *ψ*	0.9827 ± 0.0325	0.7129 ± 0.0231	<0.001
Finite predation rate, *λ*	1.2441 ± 0.0458	0.8855 ± 0.0314	<0.001
Transformation rate, *Q_p_*	0.1784 ± 0.0289	0.1495 ± 0.0191	0.3376

Significant difference within each row calculated by paired bootstrap test (TWOSEX-MSChart; *p* < 0.05).
